# Lipid droplets and the transcriptome of *Mycobacterium tuberculosis* from direct sputa: a literature review

**DOI:** 10.1186/s12944-021-01550-5

**Published:** 2021-10-03

**Authors:** Daniel Mekonnen, Awoke Derbie, Adane Mihret, Solomon Abebe Yimer, Tone Tønjum, Baye Gelaw, Endalkachew Nibret, Abaineh Munshae, Simon J. Waddell, Abraham Aseffa

**Affiliations:** 1 Department of Medical Microbiology, College of Medicine and Health Sciences, Bahir Dar University, Bahir Dar, Ethiopia; 2 Institute of Biotechnology, Bahir Dar University, Bahir Dar, Ethiopia; 3 The Centre for Innovative Drug Development and Therapeutic Trials for Africa (CDT-Africa), Addis Ababa University, Addis Ababa, Ethiopia; 4 Armauer Hansen Research Institute, Jimma Road, ALERT Compound, PO Box 1005, Addis Ababa, Ethiopia; 5 Department of Medical Microbiology, Immunology and Parasitology, College of Medicine and Health Sciences, Addis Ababa University, Addis Ababa, Ethiopia; 6 Department of Microbiology, University of Oslo, PO Box 1071, Blindern, NO-0316 Oslo, Norway; 7 Coalition for Epidemic Preparedness Innovations, CEPI, P.O. Box 123, Torshov, 0412 Oslo, Norway; 8 Department of Microbiology, Oslo University Hospital, PO Box 4950, Nydalen, NO-0424 Oslo, Norway; 9 Department of Medical Microbiology, School of Biomedical and Laboratory Sciences, College of Medicine and Health Sciences, University of Gondar, Gondar, Ethiopia; 10 Department of Biology, Bahir Dar University, Bahir Dar, Ethiopia; 11 Department of Global Health and Infection, Brighton and Sussex Medical School, University of Sussex, Brighton, BN1 9PX UK

**Keywords:** Mycobacterium, Sputum, Tuberculosis, Lipid droplet, Transcriptome, Host-pathogen interaction, Transmission, Treatment outcome, Lineage

## Abstract

*Mycobacterium tuberculosis* (*Mtb*), the main etiology of tuberculosis (TB), is predominantly an intracellular pathogen that has caused infection, disease and death in humans for centuries. Lipid droplets (LDs) are dynamic intracellular organelles that are found across the evolutionary tree of life. This review is an evaluation of the current state of knowledge regarding *Mtb*-LD formation and associated *Mtb* transcriptome directly from sputa.

Based on the LD content, *Mtb* in sputum may be classified into three groups: LD positive, LD negative and LD borderline. However, the clinical and evolutionary importance of each state is not well elaborated. Mounting evidence supports the view that the presence of LD positive *Mtb* bacilli in sputum is a biomarker of slow growth, low energy state, towards lipid degradation, and drug tolerance. In *Mtb*, LD may serve as a source of chemical energy, scavenger of toxic compounds, prevent destruction of *Mtb* through autophagy, delay trafficking of lysosomes towards the phagosome, and contribute to *Mtb* persistence. It is suggest that LD is a key player in the induction of a spectrum of phenotypic and metabolic states of *Mtb* in the macrophage, granuloma and extracellular sputum microenvironment. Tuberculosis patients with high proportion of LD positive *Mtb* in pretreatment sputum was associated with higher rate of poor treatment outcome, indicating that LD may have a clinical application in predicting treatment outcome.

The propensity for LD formation among *Mtb* lineages is largely unknown. The role of LD on *Mtb* transmission and disease phenotype (pulmonary TB vs extra-pulmonary TB) is not well understood. Thus, further studies are needed to understand the relationships between LD positivity and *Mtb* lineage, *Mtb* transmission and clinical types.

##  Introduction

### *Mycobacterium tuberculosis*

The genus Mycobacterium encompassed over 170 species and the pathogenic species are classified in to three: *Mycobacterium tuberculosis complex (MTBC: Mycobacterium tuberculosis, Mycobacterium africanum, Mycobacterium bovis, Mycobacterium microti, Mycobacterium canettii, Mycobacterium caprae), Mycobacterium leprae* and *M. ulcerans*. Among the species in MTBC, *Mycobacterium tuberculosis (Mtb*) is the main etiological agent of tuberculosis (TB) and is an intracellular pathogen that has ravaged humanity for centuries [1]. The evolutionary success of *Mtb* is attributed to its ability to flip-flop between different metabolic/phenotypic states, adaptation to diverse microenvironments, inhibition of phagolysosome fusion, and formation of necrotic granuloma [2, 3]. More than 24.8% of the global human population may harbor *Mtb* [4] in different fatty tissues [5] in the form of latent TB. Ten million active infections and 1.4 million deaths were reported in 2019 [6]. The lipid-rich sputum, and its source pulmonary granuloma microenvironment carries phenotypically heterogeneous population of *Mtb* [7].

### Lipid and lipid droplet in *M. tuberculosis*

Lipid is an inclusive term for fats and lipoids. Lipids include all of the alcohol ether soluble constituents of protoplasm such as fats, oils, waxes and several complex lipids (phospholipids, glycolipids, sulfolipids, aminolipids, chromolipids, and fatty acids) [8, 9]. Mycobacteria contain different types of structural [10, 11] and nonstructural [12] lipids. Lipids are a major source of energy [13] and play a vital role in virulence, pathogenicity, and persistence [3]. Unlike other prokaryotes, 60% of *Mtb* cell-wall constituents are lipids, mainly mycolic acids. Moreover, 40% of the dry weight of mycobacteria is derived from lipids. *Mtb* stores its chemical energy in the form of neutral lipids by forming emulsion vesicles inside the aqueous phase cytoplasm [14]. In general, lipids are the rations, attire and armor of *Mtb* [15]; as such, the diagnosis, treatment, drug resistance [16] and immunological lifecycle of *Mtb* [17] is heavily relies on membrane and/or cytoplasmic lipids.

**Fig. 1:**
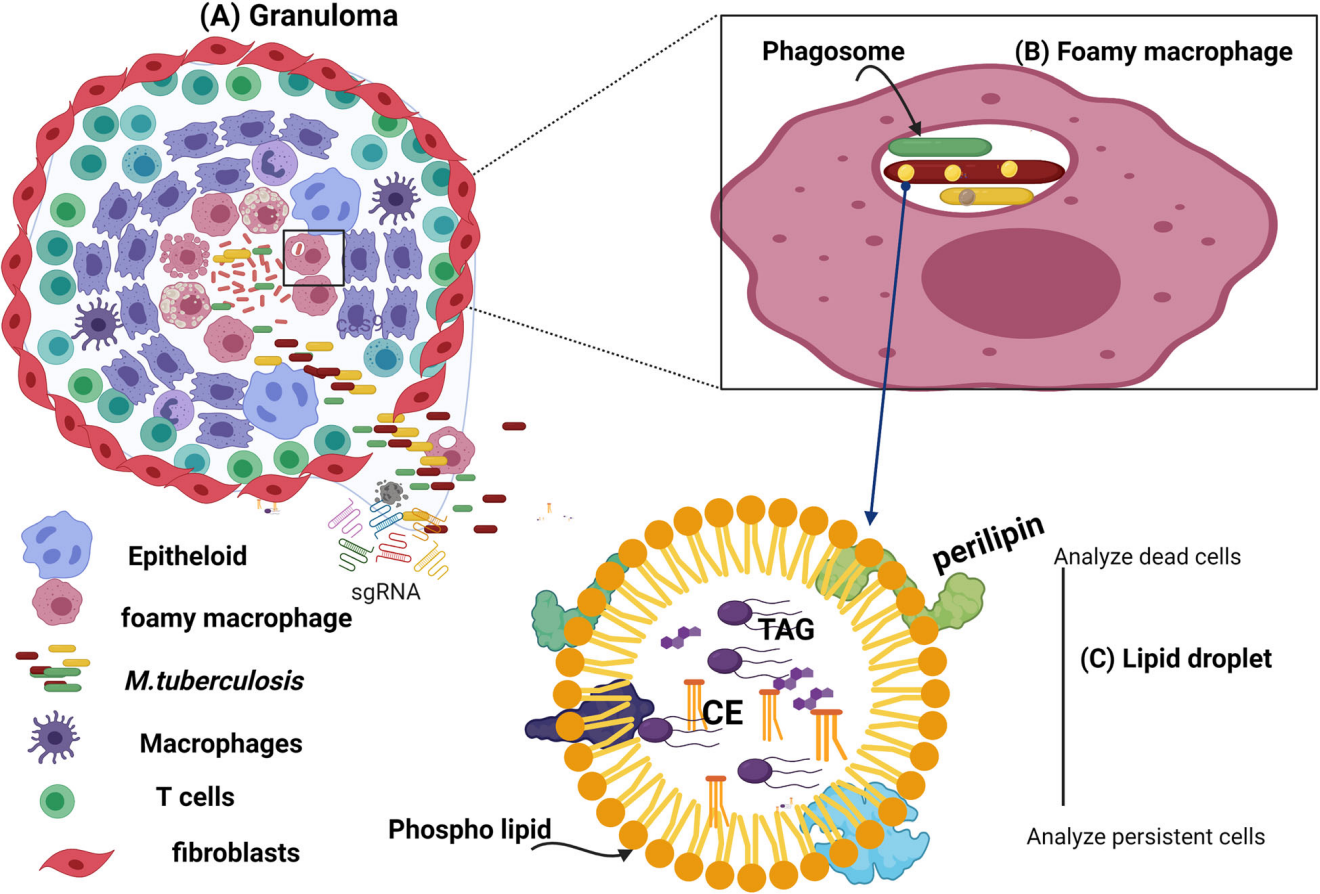
**A**:Necrotic granuloma, **B**: Foamy macrophages that contain LD-positive *Mtb* in granuloma tissue, phagocytosed *Mtb* **C**: Lipid droplet. **A:** The necrotic granuloma is a cryptic infectious immunopathological architecture and compacted collection phagocytic cells. It is the hallmark of tuberculosis [24]. Evidence showed that, except macrophages which serve as a feeder for new *Mtb* infection, innate immunity has only a little role in the initiation of granuloma formation and bacterial virulence factors such as trehalosdimycolate and ESX-1 are the driving factors for priming granuloma formation [25]. Once it is primed, dendritic cells migrate to regional lymph nodes, activate Th cells making the granuloma mature through layering of cells (macrophage, foamy macrophage, epithelioid, T cells and fibroblasts) [25]. The macrophage is the predominant phagocytic cell which occurs in differentiated forms. These are epithelioid, multinucleated giant cells, foamy macrophages and ruffled membrane macrophages [24]. *Mtb* might be found in the granuloma microenvironment due to rupture of phagosome and foamy macrophages. When the granuloma ruptures *Mtb* will be seeded to the environment through coughing, sneezing and talking. The metabolism and the level of stress in each microenvironment is different, driving *Mtb* into at least three distinct phenotypic and metabolic states; actively replicating (green), Lipid droplets (LD) loaded persister phenotype (red) and borderline between the two states (yellow). **B:** A macrophage that ingests *Mtb* through phagocytosis may harbor multiple *Mtb* phenotypes and may become a warehouse of lipid and serving as an energy reserve. These lipid-loaded macrophages are called foamy macrophages). **C:** Lipid droplets are composed of a hydrophobic core of neutral lipids (triacylglycerol, TAG and cholesterol ester, CE) surrounded by a phospholipid monolayer (phosphatidylcholine (PC), phosphatidylethanolamine (PE), phosphatidylinositol (PI) and lyso-phospholipids) decorated with different proteins. LD is an efficient energy storage organelle, as the most compacted and efficient means to store excess lipid in cells. Figures are created with BioRender.com

Cells store excess lipids inside the cytoplasm and this stored lipid is known by different names depending on the type of cells and tissue. These names includes lipid droplet (LD), lipid body (LB), intracellular lipid inclusions (ILI), oil body (OB), adiposome, spherosome and oleosome. Lipid droplets are pervasive and dynamic subcellular organelles of diverse morphological and functional diversity [18–21] across evolutionary tree of life. Lipid droplets comprised of a hydrophobic core of neutral lipids (triacylglycerol, TAG and cholesterol ester, CE) surrounded by a phospholipid monolayer of phosphatidylcholine (PC), phosphatidylethanolamine (PE), phosphatidylinositol (PI) and lyso-phospholipids; decorated with different types of proteins such as Perilipins (PLIN) [22, 23] (Fig. 1). For a detailed understanding regarding the (cell) biology and biophysics of LD, readers are advised to refer these excellent reviews [26, 27].

It was demonstrated that the LD of *Mtb* is derived from host fatty acids and that isocitrate lyase *(*encoded by *icl*) is the responsible enzyme that catabolizes fatty acids (FA) through glyoxylate cycle. Triacylglycerol synthase 1 (coded by *tgs1*) is the primary enzyme involved in triacylglycerol (TAG) synthesis and that the deletion of the *tgs1* gene led to complete loss of TAG accumulation by *Mtb* [28]. The role of sputum derived LD positive *Mtb* in treatment outcome and transmission has been demonstrated [29]. The presence of LD-positive *Mtb* bacilli in sputum is a biomarker of slow growth, low energy state, lipid degradation, anaerobic metabolism, and non-mutational drug tolerance. Sputum-derived LD-positive *Mtb* transcriptome data reveals distinct patterns of gene expression; displaying up- and down-regulation of specific metabolic pathways in sputum microenvironment. In general, the LD profile and transcriptome of *Mtb* directly from sputa are real-time metabolic, phenotypic and physiological markers of the *Mtb* population diversity and dynamics. However, the relationships between *Mtb* LD with *Mtb* lineages, *Mtb* transmission capacity and clinical pathology (i.e., pulmonary TB vs extra pulmonary TB) are not well studied or understood. We hypothesize that LD-loaded *Mtb* cells in sputum are like “rocket blast off for planned orbital mission”. Thus, this review synthesized the current state of *Mtb* LD knowledge and showed gaps for fueling future areas of research.

## Advent of *M. tuberculosis* lipid droplet research

The presence of fatty material inside the cytoplasm of prokaryotic cells was first demonstrated in 1946 by Burdon using the technique of Sudan black intracellular staining [30]. According to this classic experiment, noticeable amounts of LD were found in the majority of studied bacteria [30]. At that time, more LDs were observed in saprophytic and *Mycobacterium leprae* than in *Mtb species* [30]. With the aim of determining the precise organization of lipids in the envelope domain of living *Mycobacteria*, Christensen et al 1999 [31] developed an improved (fluorescent lipophilic probes) technique that is less disruptive than detergents [32] or ultra-sonication [33]. After probe labeling of cultivated *Mtb*, cells were observed by phase-contrast and epifluorescence microscopy. Using this technique, distinct lipid domains of *Mycobacteria* were visualized, including the envelope and LDs [31]. Generally, the lipid domains of *Mycobacteria* are comprised of three parts; the annular envelope, internal peripheral deposits contiguous with the envelope, and distinct LDs that are not associated with the envelope [31].

Following Burdon [30] and Christensen et al [31]*,* Garton and colleagues [29] advanced the field through biochemically characterizing LD in *M. smegmatis* and *Mtb*, and by analyzing factors affecting lipid formation, and the synthesis pathways in these mycobacterial species. The effects of various chemicals and growth conditions on LD were examined using Youmans’ and Middle brook 7H10 culture medias. Cells were stained with Auramine-O followed by Nile red and then stained regions were detected by epifluorescence microscope. Images were captured using a microcomputer controlled CCD camera [29]. The findings showed that, in low-carbon Youmans’ broth (YB), *M. smegmatis* showed high annular pattern and low level of LD. In contrast, in low-nitrogen YB, the annular labelling was lost and prominent LDs were observed. In addition, this study proved that LDs were formed during stationary-phase of growth. Furthermore, this study confirmed apparent indifference to carbon sources such as glucose vs glycerol on LDs formation. However, addition of exogenous fatty acids (oleic or palmitic) promoted the formation of LD, confirming the decisive role of fatty acids for *Mtb* energy systems and structural carbon sources. Further analysis identified the chemical composition of LD in *M. smegmatis*. For this, the non-polar lipids were extracted and analyzed using thin layer chromatography, Proton Nuclear Magnetic Resonance (NMR) and gas chromatography-mass spectrometry (GC-MS). The results showed that TAG was the principal component. Extending the *M. smegmatis* research above into pathogenic *Mtb* isolated from TB patients’ sputa confirmed the presence of LD in *Mtb* from sputum, and from stationary phase of cultured Mtb [29]. LD synthesis pathway analysis showed that TAG might be imported directly from macrophages or synthesized de novo [12, 13]. For a detailed understanding of LD nucleation, readers are advised to refer to excellent reviews elsewhere [34–36].

## Lipid droplets in macrophage and *Mtb* evolutionary arms race

*Mtb* can exist extracellularly in the granuloma microenvironments, or in the cytoplasm or phagosome of (foamy) macrophages (FM). Several hypotheses have been proposed regarding the survival strategies of *Mtb* inside the acidic phagosome of macrophage [37]. From these, the majority of studies support the view that *Mtb* survives inside the hostile phagosome environment by avoiding the fusion of lysosome with phagosome [38–41]. Briefly, *Mtb* avoids phagolysosome amalgamation through retaining immature phagosome markers (Rab5, Rab11 and Coronin1/TACO) and blocking the recruitment of mature endosome markers (Rab7, CD63 and Cathepsin D) at phagosome surfaces [37].

A second view suggests that phagolysosome fusion occurs, but that *Mtb* resides inside the hostile phagosome environment through upregulation of serine proteases such as Mycobacterial acid resistance Protease (MarP) [42]. MarP is an acid tolerance and virulence factor. The seminal experiment was carried out by Botella et al. (2017) to differentiate whether *Mtb* survival is via acid tolerance or phagolysosome fusion avoidance. To resolve this issue, two transposon mutants were prepared; marP::Tn (acid susceptible) and ptpA::Tn (↑lysosomal trafficking for enhancing lysosomal action). The study confirmed that, marP::Tn mutants became hypersusceptible to lysosomal content and growth attenuation occurred. Furthermore, 25 times higher attenuation rate was observed among marP::Tn (acid susceptibility) than ptpA::Tn (phagolysosome fusion) mutants. This showed that lysosomal acid tolerance was a more significant determinant than avoidance of phagosome-lysosome fusion [42]. Botella and colleagues further elaborated the mechanism of action of MarP. Accordingly, RipA, a peptidoglycan hydrolase is a substrate for MarP and acid tolerance is achieved when MarP cleaves RipA for its biological function [43].

**Fig. 2:**
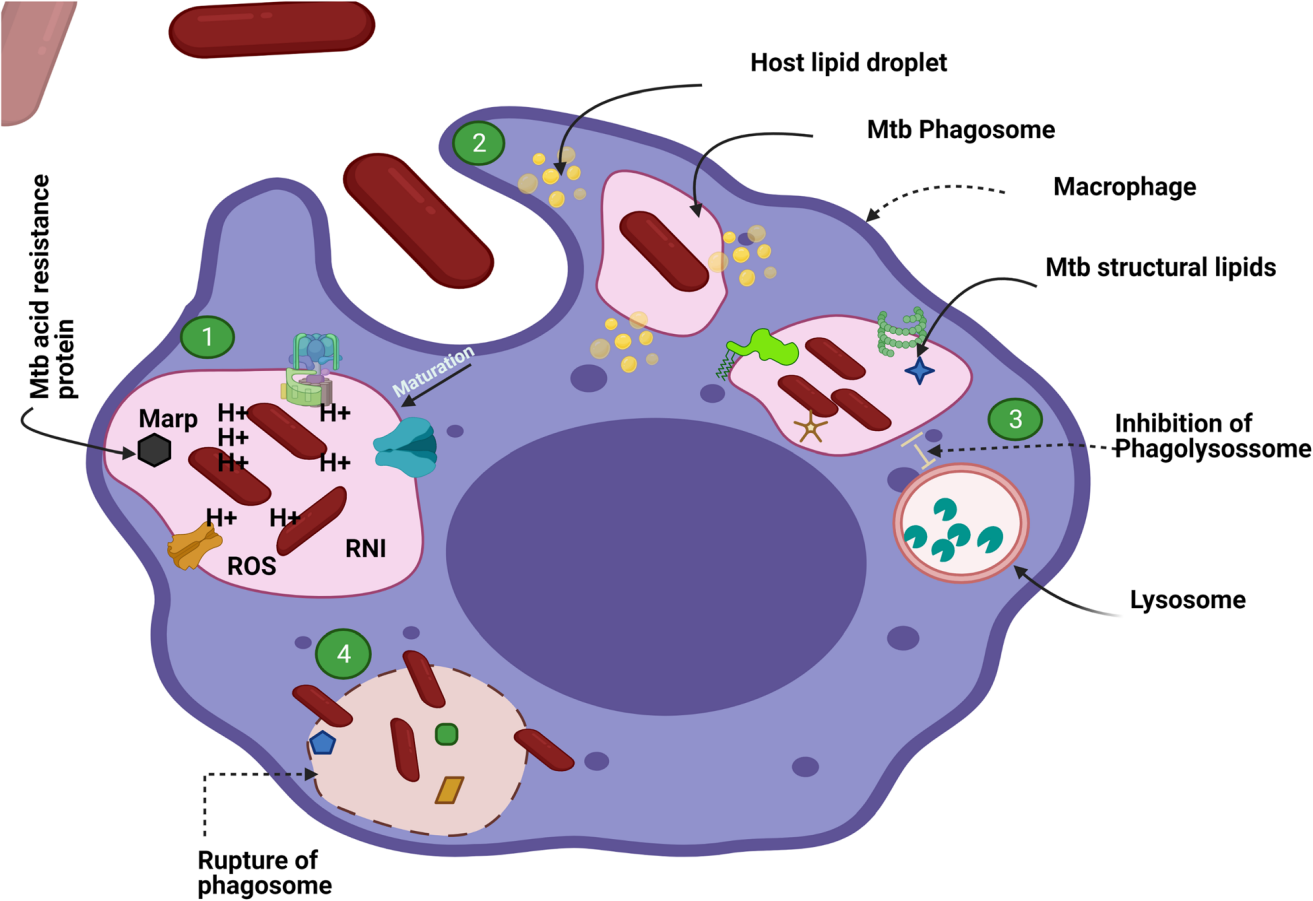
Survival strategies of *M. tuberculosis* inside the phagosome environment. This figure illustrates the mechanisms proposed to allow *Mtb* to survive inside the phagosome or *Mtb* escaping mechanisms from host defense. (1) *Mtb* survives inside the hostile phagosome by expressing Mycobacterial acid resistance Protein (MarP), a protein that buffers the acidic milieu. (2) *Mtb* survives inside the phagosome and evades the host immune response by residing apposition to the host lipid droplet. (3) *Mtb* avoids phagosome maturation and phagolysosome fusion by tagging early endosome markers (Rab5, Rab11, coronin1/TACO) and avoiding attachment and activation of several others (Rab7, CD63, lysosomal hydrolase, cathepsin D), which inhibits the proton–ATPase activity. *Mtb* accomplishes this by expressing various virulent factor lipoproteins (Man LAM, secreted phosphatase, lipid phosphatidylinositol 3 phosphate, phosphatase ptpA, TDM). (4) *Mtb* exits the phagosome and replicates inside the cytoplasm by rupturing the phagosome expressing ESX-1, DIM/PDIM, and phospho lipase A2 [46]*.* This phagosomal escape is advantageous to the pathogen for acquiring essential amino acids (arginine, methionine, or leucine), replication and dissemination [47]. *Mtb*: *M. tuberculosis*; **Man LAM**: Mannosylated lipoarabinomannan; **TDM**: Trehalose-6,6′-dimycolate; **ESX-1**: Early secretary antigenic target 6 (ESAT6) secretion system like protein; **TACO**: tryptophan aspartate containing coat protein, also named P57, Coronin1; **DIM/PDIM**: phthiocerol dimycocerosates. Figure is created with BioRender.com

The third hypothesis suggests that *Mtb* survives inside the phagosome through its interaction with host LD. Host LD helps *Mtb*-phagosome evading the macrophage’s defense systems [44]. In this survival pathway, the different *Mtb* cell wall components are participated. For instance, lipoarabinomannan (LAM) block endosome maturation and phosphatidylinositol mannosides (PIM) nourish the pathogen [44]. Additionally, the LucA protein from *Mtb* forms a complex with Mce1 and Mce4 fatty acid transporters to scavenge cholesterol and fatty acids from the cytoplasm of the macrophage [45]. The conclusion is that two or more of these survival strategies are employed by *Mtb*. Figure 2 below illustrates these *Mtb* survival strategies.

Whether macrophage LD formation is in favor of pathogen survival or part of the host defense is a subject of on-going debate. Some studies suggest that host LD gives an evolutionary advantage to the bacilli by serving as depot of chemical energy [28, 44, 48] and shelter [49, 50]. In Barisch et al (2017) review, host LD is found in close apposition to the *Mtb* phagosome, serving as a lipid supply for *Mtb* LD formation via fusion, coalescence or lipophagy-dependent internalization [48]. Peyron et al (2008) supported this hypothesis [51], showing that FM formation is a unique feature of pathogenic *Mycobacteria* (*Mtb*, *M. avium*) and oxygenated mycolic acid played a role in the differentiation of macrophages into FM. Peyron and colleagues infect macrophages with *Mtb* and scanned the formation of the granuloma at days 3 and 11 using electron microscopy. At 3-days after infection, *Mtb* was found only inside the phagosome or around the granuloma microenvironment but not inside the cytoplasm of FM. At 11-days post infection, the FM population increased from 9% (day^− 3^ post infection) to 41%, the size of LD of FM were also increased (> 5 LD/FM), and 1–20 phagocytosed *Mtb* were observed. While 60% of phagosomes were evenly distributed in the cytoplasm of FM, nearly 21% of phagosomes were stationed in close proximity to the FM-LD and progressive engulfment was observed. This study also noted that only *Mtb* that transferred from the phagosome to FM-LD became LD positive, thereby proposing that *Mtb* LD may be derived from FM-LD [51]. According to Daniel et al. (2011), hypoxia is also another key factor for macrophage LD formation, where host LD in the form of TAG were incorporated into *Mtb* LD [28]. Taken together, these studies support the view that, host and *Mtb* LDs benefit the evolutionary success of the pathogen.

In apparent contradiction to the above research, a study by Knight et al. (2017) suggested that host LD formation is entirely dependent on IFN-γ/HIF-1α activation and few LDs are observed without these cytokines. For instance, when primary murine bone marrow derived macrophages were infected with *Mtb*, very few, (average of < 1) LD were formed by macrophages. However, a large number of LD (average of > 10 LDs/macrophage) were formed when these *Mtb*-infected macrophages were treated with IFN-γ; 100% of macrophages accumulated LD. Additionally, the authors showed that *Mtb* can extricate different types of lipid from the host. Knight et al. concluded that *Mtb* LD and host LD formation are two opposing and uncoupled phenomena; where *Mtb* LD are synthesized as means of *Mtb* survival, whereas host LD synthesis is a mechanism of host defense [52]. Other studies have also reported that the accumulation of LD in the macrophage cytoplasm is part of a coordinated host defense mechanism [52–54].

The up-regulation of *Mtb* genes (*hspX, icl1, tgs1, dosR, lipY, pckA*) related to LD metabolism and hypoxia in the granuloma and inside the phagosome environment confirmed the rescue function of *Mtb*-LD [55]. Taken together, the evidence supports the view that *Mtb* LD formation serves the pathogen, and may act as a source of chemical energy [12, 56], shelter of genomic DNA [50], scavenger of toxic free fatty acids [12], creating non-mutational phenotypic heterogeneity [7] such as formation of antibiotic tolerance [55, 57–59] and evading host immune cells by hiding its pathogen associated molecular patterns (PAMP) [60, 61]. The host LD and the *Mtb* cell wall lipid components are also essential for *Mtb* survival by avoiding autophagy and delaying lysosomal trafficking towards the phagosome [62, 63].

## Clinical relevance of *M. tuberculosis* lipid droplets

### Dynamics and role of LD positive *Mtb* during TB treatment

Sloan et al 2015 hypothesized that the proportion of LD-positive *Mtb* in sputum influences the outcome of TB treatment. To address this, sputum culture and Auramine-LipidTox staining of sputum smears were carried out on consecutively collected samples through the treatment period and the treatment outcome was recorded as good or bad [64]. The study found a higher proportion of LD-positive *Mtb* among patients with poor treatment outcome [64]. Kayigire et al (2015) assessed the dynamics of LD positive *Mtb* in sputum over treatment period. The study identified three types of *Mtb* in TB patients; vegetative cells that stained positively with Auramine-O (green), LD-positive Nile-red stained *Mtb* (red) and Mtb cells stained by both (cream cells; that were borderline between the two), whose relative proportions changed over the course of treatment. Prior to the start of anti-TB treatment, green cells predominate and LD-positive cells (red) shared a small proportion of all *Mtb*. As treatment proceeded, there was a clear shift towards fewer replicating/green *Mtb* cells and more borderline and red LD-positive cells [65]. Taken together, these data suggested the value of LD staining techniques for monitoring treatment outcomes. Since LD-positive *Mtb* appear to be drug-tolerant (or drug-resistant) and refractory to staining with Auramine O, techniques targeting these phenotypes might have higher resolution and become a sensitive biomarker for treatment monitoring and predicting treatment outcome. Moreover, such studies may combine with drug discovery programs that target drug-tolerant populations of *Mtb* [66–68].

### The role of *Mtb*-LD in TB transmission

The role of LD in *Mtb* transmission is a controversial issue that warrants further scrutiny [29, 69]. Jones-López and colleagues determined the variation in *Mtb* transmission from infected households to their close contacts. The finding showed significant heterogeneity of *Mtb* transmission among human living together in a single household. This study classified isolates into *Mtb* high transmission (*Mtb*-HT) and *Mtb* low transmission (*Mtb*-LT) strains [69]. According to this study, *Mtb*-LT isolates showed an increased LD accumulation than *Mtb*-HT isolates. Moreover, in Animal model study by Verma et al, *Mtb*-LT isolates showed high growth rate. Furthermore, diffused inflammatory lung pathology, high CD8+ T cells, high inflammatory response and high mortality rate were observed among TB patients infected with *Mtb*-LT isolates. On the contrary, well defined circumscribed lesions, high degree of granuloma, caseous necrosis, cavitary lesion and high transmission rate were found among patients infected with *Mtb*-HT isolates. Hence, this study suggest that the presence of LD per se does not confer a specific transmission fitness and transmission phenotype [70], a result in contrast with the Garton et al study [29]. Collectively, TB transmission rate is deduced to be driven by several factors and TB transmission study should consider clinical presentation, host immunity, pathogen and environmental axis. Since sputum derived *Mtb* are phenotypically and metabolically heterogeneous, which phenotypes (containing differing proportions of LD positive *Mtb*, LD negative, border line or all types) are more transmissible is unknown and further study is required.

### LD-positive *Mtb* in pulmonary and extra pulmonary TB

Little is known about the relationship between *Mtb*-LD formation and clinical manifestation of disease as pulmonary TB (PTB) versus extra-pulmonary TB (EPTB). Lung is the primary site of TB disease initiation and lymph node is the primary site of adaptive immune initiation. Initiation of an adaptive immune response to *Mtb* depends on the transport of live bacteria from the lung to the mediastinal lymph nodes, and delay of this process may be advantageous for the pathogen [71]. Ganchua et al (2018) suggested that the lymph node (LN) provides an ecological niche for *Mtb*, based on evidence of higher survival of *Mtb* in the LN than in lung granuloma. This may be because granulomas that form in LNs lack B cell-rich tertiary lymphoid structures. With this, LNs are not only sites of antigen presentation and immune activation during infection, but also a niche that is protected from adaptive immune-mediated responses [72]. Severe diseases like EPTB is the outcome of a co-evolutionary mismatch [73]. The pathogen’s fitness depends on its ability to cause a high level of damage to its human host [73, 74]. Little is known about relative proportions of LD-positive *Mtb* in pulmonary parenchymal and lymph node tissue. In this regard, Maji and colleagues analyzed the transcriptome of tubercular lymphadenitis tissue and observed downregulation of host lipid metabolism related genes, in contrast to pulmonary tissue. This study confirmed the differential expression of lipid metabolic signatures between TB lymphadenitis and PTB [75]. However, the *Mtb*-LD related transcriptome and the proportion of LD positive *Mtb* among PTB and EPTB was not determined. The observation of a shift from predominant pulmonary TB to predominant LN-associated TB in certain geographic regions like Ethiopia, is provocative. The link between *Mtb* lineages and type of TB (PTB Vs EPTB) is unclear [76, 77] and some association between *Mycobacterium africanum* (MAF) and EPTB [78–80] have been reported. In general, LD formation profiling among *Mtb* and MAF isolates disaggregated by types of TB (EPTB Vs PTB) might narrow the existing knowledge gap.

### Lipid droplets formation and *Mtb* lineages

The Beijing lineage (lineage 2) of *Mtb* appears to be the slowest in time to culture conversion after the start of anti-TB treatment [81]. A study comparing the phenolic glycolipid (PGL), TAG and *dosR* regulon of Beijing lineages with lineage 3 and lineage 4 showed striking variation among lineages [82]. Briefly, while 10, 60 and 80% isolates under Beijing lineages (groups 3, 4 and 5 respectively) contain PGL, other strains from Beijing and non-Beijing lineages did not produce PGL. Additionally, while all the 36 isolates from Beijing lineages included in the analysis produced TAG, the 18 non-Beijing lineages included in the analysis failed to synthesize detectable amounts of TAG during in vitro aerobic culture [82]. However, while the authors reported 100% TAG production in Beijing lineage (L2 strains), they reported no accumulation of TAG in L3 and L4 *Mtb* isolates, a result that seems very unlikely. The culture conditions might be one factor leading to this discrepancy.

Diarra et al (2018) conducted a prospective cohort study to determine whether *M. africanum* (MAF, L6) responds faster to TB treatment more quickly than *Mtb*-L4, using Auramine O and Fluorescein Diacetate (FDA) viability stains. The authors found that MAF responded better to TB treatment but time kill kinetics was slower for MAF than L4 [81]. One would predict that slow smear conversion might lead to more transmission and drug resistance, however, based on clustering and drug resistance data, rates of transmission and drug resistance were not higher for MAF compared with *Mtb* [83, 84]. The link between poor treatment outcome, drug tolerance and LDs is well explained elsewhere [28, 64, 65]. The slow growth rate [79, 81, 85] and slow clinical recovery rate associated with MAF strains among TB cases has also been reported elsewhere [86]. Similar to MAF, *Mtb* lineage 7, which is restricted to Ethiopia and the Horn of Africa, grew more slowly in vitro and produced smaller colonies on solid media [87] in comparison to other *Mtb* strains. It is not known whether any of these characteristics correlate with LD. Collectively, the propensity of Mtb LD formation among lineages is known and a simple LD comparative analysis might provide insight regarding the differential LD formation among *Mtb* lineages.

### The proportion of LD-positive *Mtb* in sputum

The clinical relevance of LD-positive *Mtb* bacilli in sputum was first elucidated by Garton and colleagues [29, 88]. These studies concluded that the proportion of LD-positive *Mtb* in sputum lies between 3 and 86%, with 2–8 LDs/bacilli [29]. Growth rate is significantly associated with the proportion of LD-positive *Mtb* bacilli in sputum [29]. Garton and colleagues concluded that the replicating phenotypes of *Mtb* in sputum were a minor component, and LD-positive *Mtb* bacilli were predominant. This report contrasted with that of the cell culture study by Daniel et al [28]. Daniel et al characterized dynamic of *Mtb* LD formation inside the hypoxic FMs incubated under 1% O_2._ After 0, 3, and 5 days of incubation from this hypoxic state, *Mtb* recovered from FMs were stained with dual Auramine-O and Nile red staining techniques. It was found that the fraction of the *Mtb* population positive for Auramine O staining decreased from ~ 86% at day-0 to ~ 40% at day-5, while Nile Red-positive LD-positive cells increased with time from ~ 35% prior to hypoxic treatment to ~ 81% at day-5 of 1% O_2_ (hypoxia) treatment, more than two-fold increment [28]. Taken together, studies which could determine the Mtb population (LD positive, LD negative, borderline) dynamic is desirable.

## Transcriptome profile of *M. tuberculosis* from patient sputa

The spectrum of *Mtb* metabolic reprogramming is better studied through transcriptome profiling. This is because the transcriptome of sputum-derived *Mtb* provides genome-wide information on the real time metabolic state of *Mtb* populations. In addition, the state of *Mtb* metabolic reprogramming is more readily ascertained from transcriptome data than from genomic data, through quantifying the changing expression levels of *Mtb* transcripts in distinct physiological conditions. Hence, evidences on this subtopic are synthesized from the transcriptome of sputum-derived *Mtb* in comparison with the transcriptomes of *Mtb* grown in vitro culture and over the course of TB treatment. This section reviewed only original articles and the methodology of the studies are summarized in Table 1.

**Table 1:** Transcriptome profiling and validation techniques used for *Mtb* sputum transcriptomics

Comparative transcriptomics of S*Mtb*	RNA profiling method	Validation	#Transcript	Reference
S*Mtb* vs culture with 7H10 agar /7H9 broth/Dubos ^a^	Microarray	qRT-PCR	516	[29]
S*Mtb* vs culture	Microarray	qRT-PCR	557	[89]
S*Mtb* vs MAF/*Mtb*	qRT-PCR		2179	[90]
S*Mtb* vs Exponential phase of liquid culture	Dual RNA seq	Nano String	198	[91]
S*Mtb* vs Stationary phase of liquid culture	Dual RNA seq	Nano String	392	[91]
Sputum vs MGIT 460 culture	Microarray	qRT-PCR	1083	[92]
S*Mtb* at Day 3 vs S*Mtb* at day0 treatment	Microarray	qRT-PCR	109	[92]
S*Mtb* at Day7/14 vs day 0 treatment	Microarray	qRT-PCR	39	[92]
Lipid rich Dubos broth^b^ vs Dextrose rich Dubos broth^b^	RNA seq	qRT-PCR	–	[60]
S*Mtb*/ BAL-*Mtb* vs 7H9/ DTA^c^ culture	qRT-PCR		–	[93]
S*Mtb* before Rx vs S*Mtb* after Rx	qRT-PCR	qRT-PCR	2411	[94]
Sputum *Mtb* vS culture H37Rv	Microarray	qRT-PCR	–	[95]

^a^7H10 agar with oleic acid-albumin-dextrose-catalase supplement or in 7H9 broth with albumin-dextrose-catalase supplement, 0.2% glycerol and 0.05% Tween-80. Hypoxic (non-replicating persistence) cultures M. tuberculosis strains H37Rv and CH were grown in Dubos Tween albumin broth. ^b^Dubos broth (Difco), without glycerol, containing 0.5% albumin, supplemented with either 0.2% dextrose or a lipid mixture (oleic acid, palmitic acid, stearic acid, at final concentration of 0.001% each, plus 0.01% cholesterol). ^c^7H9 media (0.05% Tween 80, 0.2% glycerol, 10% ADC supplement)/ DTA: Dubos Tween albumin; for the NRP-2 model was grown in 100 mL Dubos Tween albumin (DTA). *SMtb* sputum-derived M. tuberculosis, *Mtb* Mycobacterium tuberculosis, *vs* versus, *Rx* treatment, *MAF* Mycobacterium africanum, *L4* Lineage 4, *qRT-PCR* Real-Time Quantitative Reverse Transcription PCR, *RNA seq* RNA-sequencing, *DTA* Dubos Tween, *BAL* Broncho alveolar lavage

The key findings of the individual studies referred in Tables 2. The transcriptome data showed distinct transcriptome profiles which might be explained by differences in the technique, study populations and number of genes targeted. The sputum-derived *Mtb* transcriptome relatively mirrored the lung/ broncho alveolar lavage (BAL) derived *Mtb* transcript profile. The slight differences between the two (sputum and BAL) might be due to the higher hypoxic state of the lung than upper respiratory tracts such as bronchi and oral cavity. Hence, sputum *Mtb* transcriptome profiling might be a substitute for the BAL transcriptome for assessing *Mtb* pathogenesis and treatment conditions [93]. Comparing the *Mtb* transcriptome in lipid and dextrose rich medium did not showed significant differential expression [60]. The sputum-derived *Mtb* transcriptome is quite different from exponentially growing *Mtb* in animal models and in-vitro.

**Table 2:** The summary of transcriptomes of *M. tuberculosis* in sputum versus other conditions, 2021

Transcriptome condition	In vitro comparator	URG in sputum *Mtb*	DRG in sputum *Mtb*	Ref
S*Mtb* vs Culture	7H10 agar /7H9 broth/Dubos^a^	*dosR*, *icl1*, *hspX*, *narK2*, *tgs1, PE/PPE*	*nuoB, qcrC, and ctaD*	[29]
S*Mtb* Vs Culture	No information	Conserved Hypotheticals. *mprAB: dosR* is stable	*pks15/1, pks10* *Pks12*, *phoP, ESX 1-ESX-5*	[89]
S*Mtb* Vs Culture	Liquid culture	ACOD1/IRG1, GLUT1, *MCT4*, *ESX-3*, Rv0106, Rv2990c, *ESX-3*, *mprA*	TCA cycle, ETC, NADH dehydrogenase, pentose phosphate pathway (PPP), NAPDH, ROS. PhoP, small RNA mcr7, *pks12*, *esat-6* and *cfp-10*, *phoP*	[91]
S*Mtb* Vs Culture	MGIT 460	Glyoxylate shunt, methylcitrate cycle (*icl, prpC and rv1129c*), catabolism of cholesterol and fatty acids, and *tgs;* Nitrate reduction (*narK2/3*). *dosR nrdZ, narK2, rv1738, pfkB, hspX, hrp1,rv3126c and rv3128c*	*gltA2, kgd, mdh, korA/B, sucC, rv0247c/48c, fumC and mqo), FAS-1 (fas), FAS-II, mmaA2/3/4, cmaA2, pcaA, fadD32 and pks13,* NADH dehydrogenase, cytochrome C reductase	[92]
S*Mtb* Vs culture	7H9/ DTA^c^	sputum and BAL had significant up-regulation of the *dosR* regulon	Ribosomal genes and primary metabolism genes	[93]
Day 7–14 days Vs day0	None applicable	Anaerobic respiration, *PE/PPE* genes, *is, dosR*,transcriptional factors, oxidative stress, sigma factors, toxin-antitoxin modules,	TCA cycle, ATP synthesis, ribosomal proteins, *pks*, *ESX*, replication, efflux pumps, drug-activating enzymes & drug targets	[94]
S*Mtb* at day 3/7/14 Vs day 0 Rx	None applicable	*Mtb* responses at 7 and 14 daysduring chemotherapy were most similar to that of bacillibefore drug therapy had begun	methylcitrate	[92]
Day14 Vs day2	None applicable	*tgs,* and ATP-binding cassette transporter and toxin. *Rv1258c, bacA*, and *mmr*, *rpoB*. TA modules, sigma factors	ESX and ribosomal genes, drug-activating enzymes katG, pncA, and ethA, gyrase, bedaquiline target *atpE*	[94]
BAL vs sputum	7H9/ DTA^c^	*dosR* regulon expression was higher in BAL than in sputum	BAL had lower expression of ribosome proteins	[93]
Lipid-NRP1 Vs Dextrose-NRP1	Dubos broth^b^	Higher virulence, detoxification & adaptation, lipid metabolism, intermediary metabolism& respiration, regulatory protein	Insertion sequences & phages	[60]
In NRP-2 state	7H9/ DTA^c^	*dosR* regulon, oxidative stress responses, anaerobic respiration	Growth and metabolism	[93]

The list of genes up /down regulated is not exhaustive, only common genes listed

^a^7H10 agar with oleic acid-albumin-dextrose-catalase supplement or in 7H9 broth with albumin-dextrose-catalase supplement, 0.2% glycerol and 0.05% Tween-80. Hypoxic (non-replicating persistence) cultures M. tuberculosis strains H37Rv and CH were grown in Dubos Tween albumin broth. ^b^Dubos broth (Difco), without glycerol, containing 0.5% albumin, supplemented with either 0.2% dextrose or a lipid mixture (oleic acid, palmitic acid, stearic acid, at final concentration of 0.001% each, plus 0.01% cholesterol). ^c^7H9 media (0.05% Tween 80, 0.2% glycerol, 10% ADC supplement)/ DTA: Dubos Tween albumin; for the NRP-2 model was grown in 100 mL Dubos Tween albumin (DTA). *SMtb* Sputum Mtb, *Mtb* Mycobacterium tuberculosis, *URG* Up regulated genes, *DRG* Down regulated genes, *BAL* Broncho alveolar lavage, *NRP* None replicating persistent state, *Vs* versus, *Rx* treatment, *dosR* Dormancy survival regulator, *hspX* a-crystallin homologue, *narK2* nitrate/ nitrite transporter, *qcrC* cytochrome bc1 complex, *ctaD* aa3-type cytochrome c oxidase, *icl1* isocitrate lyase gene, *nuoB* type-I NADH dehydrogenase, *Pks12* Polyketide synthase, *PDIMs* Phthiocerol dimycocerosates, *PGLs* phenolic glycolipids

Relative to pretreatment expression, the mRNA abundance decreased by 50% over 12 h during the first 2 days of anti-TB treatments shots [94]. Over the course of anti-TB treatment, genes encoding drug activating enzymes such as a catalase peroxidase (*katG*), nicotinamidase/pyrazinamidase (*pncA*), and Ethionamide activator (*ethA*) showed repression, indicating that majority of the *Mtb* populations are dying and entered into drug related stress tolerance state [94]. Genes related to persister phenotypes such as triacylglycerol synthases and, ATP-binding cassette transporter and toxin molecules were induced [94].

In terms of energy utilization, the ATP synthase operon in sputum was downregulated and the transcriptome of sputum-derived *Mtb* was more similar to the transcriptome of *Mtb* during stationary phase growth than during exponential growth of *Mtb* in-vitro. Decrease in abundance of *phoP* and *esx* transcripts indicated a switch to lipolysis and decreased virulence [91]. PhoPR a two-component system is essential for virulence through its secretary function and its mutation leads to a loss of virulence [96]. Because the *Mtb* in sputum has originated from a granuloma rich in lipid, it is not unexpected that the transcriptome of sputum-derived *Mtb* microenvironment is enriched for transcripts involved in lipid metabolism [91, 92], microaerophilic respiration, low energy state, and persistence [29, 94].

The DosR regulon (*dosR*) which constitutes over 50 genes [4] is activated by low oxygen tension [97] and accumulation of oxygen byproducts such as H_2_O_2,_ CO, NO, and ethanol [98]. The *dosR* regulon is over expressed in sputum and during anti-TB treatment [92, 94] compared with log phase in vitro culture [92, 93]. The expression of the *dosR* regulon is likely a general indicator of bacteria’s tolerance to oxygen and may have no direct role in LD metabolism. Hence, while expression of the *dosR* regulon is observed in both growing and persister populations of *Mtb* [29, 99], its expression is dependent on the degree of the hypoxic state. Comparatively upregulation of *dosR* regulon was observed among Lineage 2 *Mtb* than Lineage 4 *Mtb* from sputum [91] and among *Mtb*-L4 than among MAF-L6 [90]. These results suggest that upregulation of the *dosR* regulon is an indicator of the aerophilic state of *Mtb*/MAF rather than a marker of metabolic states linked to LD.

*M. tuberculosis* genes such as *dosR* regulon, *hspX*, *mprAB* and *PE/PPE* and those involved in the glyoxylate shunt, methylcitrate cycle, cholesterol catabolism, nitrate reduction metabolism were upregulated relative to log phase control H37Rv cells grown in vitro [29, 89, 91, 92]. In contrast, the tricarboxylic acid (TCA) cycle, electron transport chain (ETC), polyketide synthase, *ESX* secretion apparatus*,* mycolic acid synthesis, NADH dehydrogenase and cytochrome c reductase were downregulated in sputum-derived *Mtb* compared to log phase aerobic in vitro culture [91, 92]. During treatment, downregulation of *ESX* secretion and anti-TB drug activating enzymes were noticed compared with pre-treatment sputum *Mtb* [94]. When under extreme stress, NRP-2 state, anaerobic respiration and *dosR* were upregulated and genes involved in growth and metabolism were repressed [93] (Table 2). Garcia et al concluded that the transcriptomes of BAL and sputum-derived *Mtb* reflect a moderate level of hypoxia approximately midway on a spectrum of the hypoxic state between aerobic growth and NRP-2 [93]. En masse, the majority of genes from the information pathway, cell wall and cell processes, virulence, detoxification, adaptation, secretion, transport, intermediary metabolism and respiration [89] are repressed in *Mtb* from direct sputum.

Anti-TB treatment is typically monitored by microscopy and culture conversion. However, such techniques are inadequate for the detecting non-replicating drug-tolerant *Mtb*, which is important for predicting treatment duration, treatment outcome and drug resistance. Techniques measuring 16 s rRNA or pre-rRNA promise to add new depth to our understanding of the efficacy of drug combinations in patients [100, 101]. Demirci and colleagues assessed the diagnostic accuracy of *Mtb*-mRNA-based RT-qPCR technique, with the BACTEC MGIT 960 method used as the gold standard. The findings were encouraging, in that the mRNA-based method appeared to be more sensitive and specific than other methods [102]. However, additional information is needed before this technique can be translated into clinical practice. In particular, questions with regard to *Mtb* persistence and dormancy need to be addressed and defined [12, 29, 103–105].

## Study strength and limitations

This review summarized pertinent information regarding the role of *Mtb* LD on host-pathogen interactions, diagnosis, treatment and transmission. As such, the review highlighted conflicting reports and advised future research areas. However, our literature search strategy is not complete and the quality of included articles were not appraised.

## Conclusions

The role of LD in the co-evolutionary arms race, granuloma formation, and treatment outcome of TB must be recognized. The power of LD in determining the distinct metabolic, physiological, phenotypic state from sputum-derived *Mtb* is described. The presence of LD is observed universally in prokaryotes including *Mtb*. However, LD are more common and more abundant: 1) in BAL-derived *Mtb* than in sputum-derived *Mtb*, 2) after anti-TB drug therapy, and 3) during stationary phase than exponential phase of growth in vitro culture.

LD are a source of chemical energy and phenotypic heterogeneity. They can also delay lysosomal trafficking towards phagosomes, block autophagy, promote immune cell evasion and scavenge toxic metabolites and signaling. The influence of LD on transmissibility and virulence of *Mtb* is less well understood. Multiple factors from the pathogen, host and environment axis might influence *Mtb* transmission, however some evidence links overproduction of LD in the *Mtb* Beijing lineage 2 and some *Mtb* lineage 4 isolates with higher transmissibility/virulence.

Several different transcriptome profiles were detected in LD-positive *Mtb*, which could reflect sample-to-sample variation, differences in methodology or other experimental conditions. Nevertheless, we conclude that *Mtb* in sputum exists in a variable phenotypic and metabolic states. The dynamics of gene expression in LD-positive *Mtb* from sputum provides clinically-important information on the evolution and pathogenicity of *Mtb*. Further studies are needed to investigate the relationships between intracellular LDs and *Mtb* lineages, *Mtb* transmission capacity, clinical phenotype and *Mtb* pathophysiology.

Transcriptomic analysis of sputum-derived LD-positive *Mtb* cells could prove to be useful in clinical and research settings. For instance, lipophilic staining targeting LD-positive *Mtb* might be more sensitive and specific than current methods, such as ZN/ FM microscopy, which only detects actively growing *Mtb*. Finally, lipid metabolism-associated genes are upregulated in LD-positive *Mtb*. Based on this observation, it may be possible to develop an mRNA based diagnostic test that is sensitive and specific for the detection of LD-positive *Mtb*. Such a test could be valuable for TB diagnostics and to monitor treatment of TB. This exciting possibility will be explored in future research.

## Data Availability

All data generated or analyzed during this study are included in this published article.

## Abbreviations

BAL: Broncho alveolar lavage
CE: Cholesterol ester
DIM/PDIM: Phthiocerol dimycocerosates.
DTA: Dubos Tween
EPTB: Extra-pulmonary TB
ESAT6: Early secretary antigenic target 6
ESX-1: ESAT6 secretion system like protein
ETC: Electron transport chain
FDA: Fluorescein Diacetate
FM: Foamy Macrophages
GC-MS: Gas chromatography-mass spectrometry
ILI: Intracellular lipid inclusions
L4: Lineage 4
LAM: Lipoarabinomannan
LB: Lipid body
LD: Lipid droplet
LDs: Lipid droplets
LN: Lymph node
MAF: *Mycobacterium africanum*
MAF: *Mycobacterium africanum*
Man LAM: Mannosylated lipoarabinomannan
MarP: Mycobacterial acid resistance Protease
Mtb: *Mycobacterium tuberculosis*
*MTBC*: *Mycobacterium tuberculosis complex*
NMR: Proton Nuclear Magnetic Resonance
OB: Oil body
PC: Phosphatidylcholine
PE: Phosphatidylethanolamine
PGL: Phenolic glycolipid
PI: Phosphatidylinositol
PIM: Phosphatidylinositol mannosides
PTB: Pulmonary TB
qRT-PCR: Real-Time Quantitative Reverse Transcription PCR;
RNA seq: RNA-sequencing
Rx: Treatment
SMtb: Sputum-derived *M. tuberculosis*
TACO: Tryptophan aspartate containing coat protein
TAG: Triacylglycerol
TB: Tuberculosis
TCA: Tricarboxylic acid cycle,
TDM: Trehalose-6,6′-dimycolate
vs: versus
YB: Youmans’ broth
